# Awareness and use of genetic testing: An analysis of the Health Information National Trends Survey 2020

**DOI:** 10.1016/j.gim.2022.08.023

**Published:** 2022-09-22

**Authors:** Jessica C. Tiner, Leah E. Mechanic, Lisa Gallicchio, Elizabeth M. Gillanders, Kathy J. Helzlsouer

**Affiliations:** 1 Epidemiology and Genomics Research Program, Division of Cancer Control and Population Sciences, National Cancer Institute, National Institutes of Health, Bethesda, MD

**Keywords:** Ancestry, Awareness, Cancer, Genetic testing

## Abstract

**Purpose:**

Genetic testing is a tool used in a variety of settings for medical and nonhealth related purposes. The goal of this analysis was to better understand the awareness and use of genetic testing in the United States.

**Methods:**

Data from the 2020 Health Information National Trends Survey 5 cycle 4 were used to assess the awareness and use of genetic testing by demographic characteristics, personal cancer history, and family cancer history.

**Results:**

Overall, 75% of participants were aware of genetic testing and 19% of participants had genetic testing. Ancestry testing was the most common type of testing that the participants were aware of and had received. Non-Hispanic Asian, Non-Hispanic Black, and Hispanic respondents and participants with incomes less than $20,000 were less likely to be aware of and have received any type of genetic testing than the Non-Hispanic White participants and participants with higher income, respectively. Participants with a family history of cancer were more likely to be aware of cancer genetic testing than those without, and participants with a personal history of cancer were more likely to have had cancer genetic testing.

**Conclusion:**

It appears awareness of genetic testing is increasing in the United States, and differences in awareness persist by race/ethnicity and income.

## Introduction

Genetic testing, which assesses the genetic variations inherited from each parent (germline variation), has multiple potential applications. In health care settings, genetic testing is used for predicting and managing the risk of developing several disease conditions, such as certain cancers, heart diseases, and a variety of inherited diseases. Genetic testing is commonly used in prenatal settings for preconception and prenatal screening. Results from these tests can have substantial effects for both individuals and their families. In addition to clinical settings, genetic testing for personal use has become increasingly popular. These tests can be used to determine ancestry, characteristic testing (eg, type of ear wax), and identifying certain health-related traits (eg, genetic variants associated with risk of specific health conditions, such as diabetes and late-onset Alzheimer disease).

The Health Information National Trends Survey (HINTS) is a nationally representative survey of adults aged 18 years and older in the United States and is designed to assess public awareness and use of health-related information. Previous research using the 2017 HINTS estimated that 57% of Americans were aware of genetic testing.^1^ Despite almost half of the Americans reporting being aware of genetic testing, awareness appears to differ across communities in the United States. Previous works have shown differences in awareness of genetic testing by race and ethnicity and income^1–4^ and differences in uptake specifically for cancer genetic testing by race and ethnicity.^5,6^

Understanding the differences in awareness is important because lack of awareness of genetic testing represents one potential barrier to receiving appropriate genetic testing. Those who are aware of genetic testing may be more interested in undergoing genetic testing and discuss it with their health care provider.^7^ Because of this relationship between awareness and receipt of genetic testing, it is critical to better understand public’s awareness of genetic testing. Differences in awareness of genetic testing may exacerbate existing disparities in health outcomes for low income and racial and ethnic minority groups.

The purpose of this study was to examine the association between current state of awareness and use of a variety of genetic tests and demographic characteristics, including race and ethnicity, family history of cancer, and personal cancer history.

## Materials and Methods

### Study sample and survey

Data were analyzed from the 2020 HINTS 5 cycle 4 study, a probability-based nationally representative survey of adults aged 18 years and older in the United States designed to assess public awareness and use of health-related information. HINTS 5 cycle 4 was a self-administered mailed questionnaire. The HINTS instrument, methodology, and data are accessible to the public for analysis online.^8^ There were 3865 respondents to the HINTS 5 cycle 4, with a response rate of 37%. The analytical sample for this study included the 3767 participants who responded to at least one of the genetic testing questions (section F) on the HINTS. The 98 respondents who were excluded from the analysis were significantly more likely to be older, not employed, and widowed/divorced/separated than those who were included in the analysis; however, they did not differ significantly in terms of race, ethnicity, income, or personal/family cancer history.

### Variable operationalization

Participants were asked separately whether they had ever heard of or had specific types of genetic tests (questions F1 and F3). For each question, participants were told to “mark all that apply” with the following response options: ancestry (to determine geographic/ethnic origin of individual’s ancestors), genetic health-risk testing (to determine health risk for variety of health conditions, eg, 23andMe), high-risk cancer testing (eg, *BRCA 1/2* or Lynch syndrome), not sure, and/or other. For the applicable outcome variable (heard of or received genetic testing), participants who selected either ancestry, genetic health-risk testing, high-risk cancer testing, or other were categorized as heard of or had any genetic test overall.

Individuals who selected “other” were asked to specify (in free text) what “other” type of test they had heard of or had received. Each “other” free text response was reviewed to determine whether the response belonged to one of the prespecified answer choices (eg, ancestry), should remain in the “other” category (eg, paternity testing), or was not a genetic test. Participants who were missing a response to heard of genetic testing (F1) and reported having a genetic test (F3) were recoded as hearing of a genetic test. Respondents who responded “not sure” for heard of genetic testing (F1) and “not sure” for having a genetic test (F3) were recoded as not hearing of/having genetic testing. Participants missing for having a genetic test (F3) who reported not hearing of a genetic test (F1) were recoded as not having a genetic test (F3). In this analysis, “hearing” about genetic testing was interpreted as awareness of genetic testing.

Demographic variables of interest included sex, race/ethnicity, age, income level, employment level, and marital status and were categorized on the basis of self-report from survey responses. A combined race and ethnicity variable was created with the following categories Non-Hispanic White, Non-Hispanic Black, Non-Hispanic Asian, Non-Hispanic other and Hispanic. Because of the small number of responses, respondents who identified as Alaska Native/American Indian, Pacific Islander, or as multiple races were combined into an “other” race category for the analysis. The counts for each of these groups are reported separately in Table 1. The combination of Alaska Native/American Indian, Pacific Islander, and multiple races into one “other” category did not affect the results of this analysis. Participants who responded that they were “Hispanic, Latino/a, or Spanish origin” were categorized as “Hispanic.” For employment, we analyzed the multiple different categories and combined participants who were retired, disabled, students, homemakers, or other into one “not employed” category because of low cell counts. Participants who reported being employed full or part time were categorized as “employed.” Categorizing in this manner did not change the results of this analysis. For marital status, the “other” category included participants who were widowed, divorced, or separated. In addition, cancer history variables were a self-reported personal history of cancer (yes/no) and having a family history of cancer (yes/no). A family history of cancer was defined as reporting a cancer diagnosis in any first- or second-degree biological relative. If a participant reported having a personal history of cancer, he or she was asked to select the type(s) of cancer he or she had ever been diagnosed with from a list of 22 common cancers. A participant could also select “other” type of cancer, if “other” was selected, the participant was asked to specify. Those who selected “skin cancer only” were recoded as not having a personal history of cancer.

### Statistical analysis

Associations between the demographic and cancer history variables with awareness of or had genetic testing were examined using multivariable logistic regression. Odds ratios (ORs) and 95% CI were calculated for each variable, adjusting for the other variables in the model. For awareness of and had high-risk cancer genetic testing, additional analyses were conducted to calculate adjusted ORs for participants with a personal history of breast or ovarian cancer (*N* = 116: *n* = 105 with breast cancer and *n* = 11 with ovarian cancer) compared with those without a personal history of cancer because there were sufficient numbers to examine this subgroup, and there are clinical guidelines for discussing genetic testing. In addition, awareness of and receipt of genetic tests were examined looking at subgroups of those with and those without a personal history of cancer and those with and those without a family history of cancer. The results of these analyses were similar to the overall analysis. In compliance with the HINTS analytical recommendations, data were analyzed using the final sample weight to obtain population-level point estimates and the set of jackknife replicate weights to obtain correct started errors.^9^ A comparison of the weighted and unweighted percentages can be found in Supplemental Table 1. A 2-tailed *P* value of <.05 was considered statistically significant. All analyses were performed in STATA/SE 16.1 (StataCorp).

## Results

**Figure 1:**
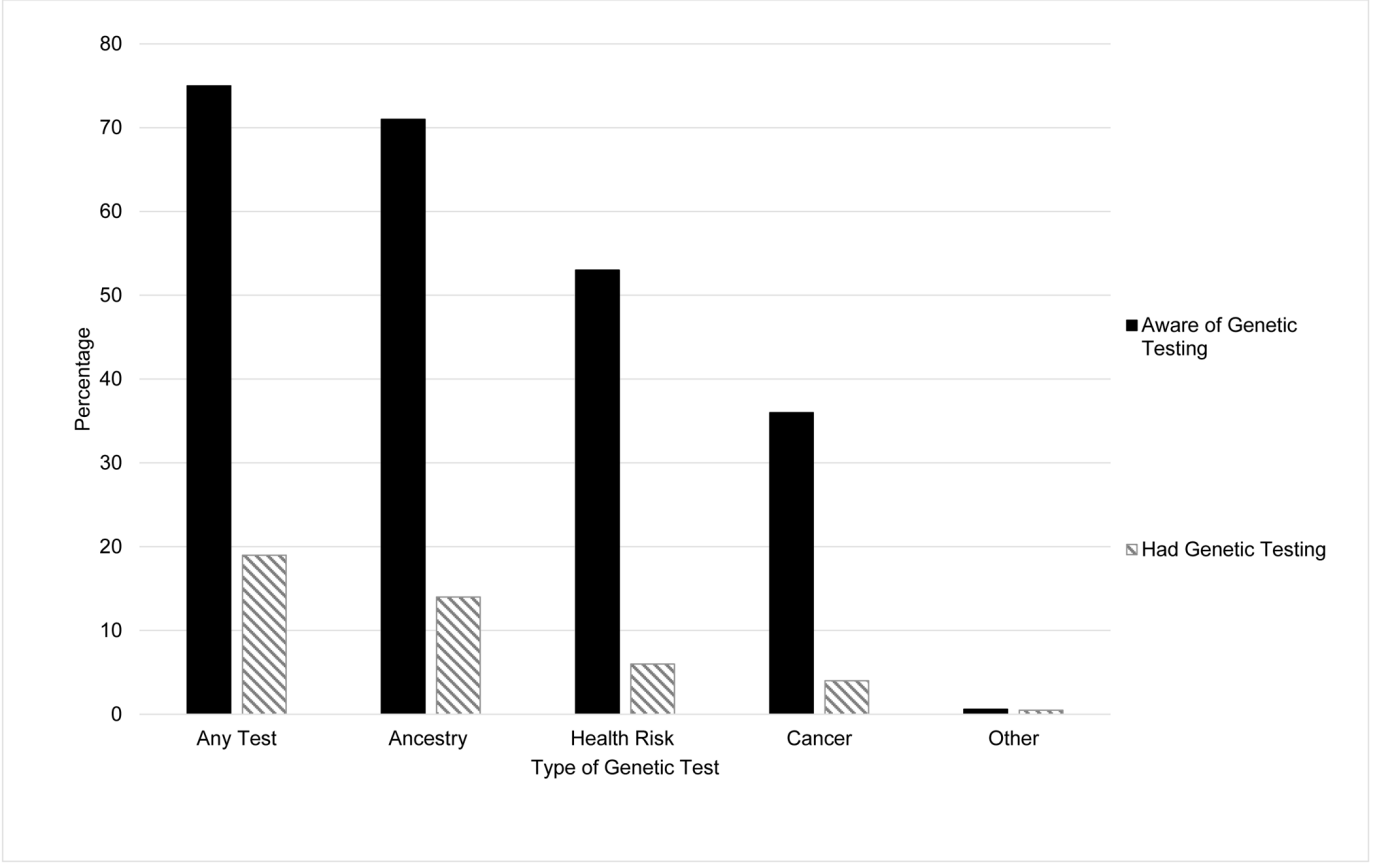
Distribution of response to awareness of or receipt of genetic testing overall and by different test types. Percentage of respondents who were aware of genetic testing (filled bars) and percentage of respondents who ever had genetic testing (hatched bars) for the type of genetic tests: “any test” or “ancestry,” “health risk,” “cancer,” or “other.”

The characteristics of the study sample are shown in Table 1. Of the respondents included in this analysis, 51% were women, and almost half were aged 50 years and older. Most participants were Non-Hispanic White (60%), had health insurance (91%), had some college education (70%), and made $50,000 or more annually (61%). In addition, 70% of participants reported a family history of cancer and 7% of participants reported a personal history of cancer. Of those with a personal history of cancer, breast (19%) and prostate (9%) were the most common types of cancer reported. Overall, 75% of participants reported hearing of any type of genetic testing. In total, 19% of participants responded having some form of genetic testing. The most common type of test participants were aware of (71%) and had (14%) was ancestry testing (Figure 1).

The results for the multivariable adjusted analysis for awareness and receipt of genetic testing are displayed in Table 2. Older, Non-Hispanic Asian, Non-Hispanic Black, and Hispanic participants were less likely to be aware of genetic testing than their younger, Non-Hispanic White counterparts, respectively (OR = 0.4, 95% CI = 0.2–0.8; OR = 0.2, 95% CI = 0.1–0.5; OR = 0.6, 95% CI = 0.4–1.0; OR = 0.4, 95% CI = 0.3–0.7, respectively). Respondents with a high school education or less and those earning less than $20,000 annually were less likely to be aware of genetic testing than those with some college education and those who had an annual income of $50,000 or more. Consistent with the results of awareness of a genetic test, Non-Hispanic Black and Hispanic participants were less likely to have genetic testing than their Non-Hispanic White counterparts (OR = 0.5, 95% CI = 0.3–0.9; OR = 0.4, 95% CI = 0.2–0.6). Females and those with an income higher than $50,000 were more likely to have had genetic testing than males and those with an income less than $20,000.

The adjusted multivariable results for awareness of a specific type of genetic test are shown in Table 3. Non-Hispanic Black and Hispanic participants were less likely to be aware of each type of genetic test than the Non-Hispanic White participants (Non-Hispanic Black: OR = 0.6, 95% CI = 0.4–0.9; OR = 0.6, 95% CI = 0.4–0.9; OR = 0.5, 95% CI = 0.3–0.8; Hispanic: OR = 0.4, 95% CI = 0.3–0.7; OR = 0.4, 95% CI = 0.2–0.7; OR = 0.5, 95% CI = 0.3–0.8). Non-Hispanic Asians were less likely to be aware of ancestry and health-risk genetic testing than the Non-Hispanic White participants (OR = 0.2, 95% CI = 0.09–0.4; OR = 0.4, 95% CI = 0.3–0.8). Respondents with a high school education or less or those who earn less than $20,000 annually were less likely to be aware of each of the genetic test types than those with some college education and those who make more than $50,000. Females were more likely than males to be aware of health related and cancer genetic testing. Participants with a family history of cancer were more likely to be aware of cancer genetic testing than those without a family history of cancer (OR = 1.8, 95% CI = 1.3–2.5).

Table 4 shows the results of the multivariable logistic regression for having specific types of genetic tests. Non-Hispanic Black participants were less likely to have ancestry testing than Non-Hispanic Whites (OR = 0.5, 95% CI = 0.3–0.9). Consistent with the results for awareness of specific types of genetic tests, Hispanic participants were less likely to have had each type of genetic testing than Non-Hispanic White participants. Participants with some college education and a higher income were more likely to have had ancestry testing than those with less than a high school education or a lower income (OR = 2.3, 95% CI = 1.5–3.3; OR = 2.3, 95% CI = 1.2–4.2; OR = 2.65, 95% CI = 1.54–4.64). Similar to the results for awareness of genetic testing, compared with males, females were more likely to have health risk and had cancer genetic testing. Participants with a personal history of cancer were more likely to have cancer genetic testing than those without a personal or family history of cancer (OR = 5.3, 95% CI = 2.7–10.3). Participants with a personal history of breast/ovarian cancer were found to be more likely to be aware of (OR = 4.7, 95% CI = 2.5–8.8) and had (OR = 36.8, 95% CI = 17.8–75.6) cancer genetic testing than the individuals without a personal history of cancer.

## Discussion

The results of this nationally representative survey of adults showed that most respondents were aware of genetic testing (75%); however, only 19% of reported having genetic testing. The most common type of genetic test that participants were aware of and had received was ancestry testing. Furthermore, this study identified lower awareness and use of genetic testing in Non-Hispanic Asian and Non-Hispanic Black participants, Hispanic participants, and individuals with an annual income less than $20,000. Participants with a family history of cancer were more likely to be aware of cancer genetic testing and participants with a personal history of cancer were more likely to have received cancer genetic testing than their counterparts without a family or personal history of cancer.

Compared with the results of a previous HINTS mailed in 2017, it appears that awareness of genetic testing is increasing, from 57% of respondents reporting that they were aware of genetic testing in 2017 to 75% in 2020.^1^ In the 2014 HINTS, 38% of respondents were aware of “genetic tests that analyzey our DNA, diet, and lifestyle for potential health risks are currently being marketed by companies directly to consumers.”^7^ Comparing the results of this analysis to the HINTS in 2017 and 2014, it appears that genetic testing awareness has increased over time. However, despite the high awareness of genetic testing in 2020, this study identified a lower percentage of respondents being aware of genetic testing for health risk and cancer purposes compared with ancestry genetic testing. Because of the high number of respondents being aware of ancestry testing, the change in awareness of genetic testing could be attributed to an increase in advertisements of at-home genetic testing kits.^10^ More work is still needed to better educate the public on genetic testing for health and cancer purposes.

Consistent with findings from previous HINTSs, this study found racial, ethnic, and income differences in awareness of genetic testing.^1^ These differences are persistent across the different types of genetic tests. Racial, ethnic, and income disparities have previously been reported in awareness of genetic testing,^3,4,7,10–15^ and these differences have remained consistent over time. Differences in awareness of genetic testing represent 1 potential barrier for receiving genetic testing and may further exacerbate health disparities for racial and ethnic communities. In addition to being aware of the test, other barriers previously reported included concerns about effect of findings for family members, the potential for negative emotional responses, and mistrust and concerns over misuse.^14,16,17^ Although these groups are experiencing multiple barriers to genetic testing, previous work has suggested that racial and ethnic minority groups are interested in participating in genetic testing.^16,18^ Because germline genetic testing may have significant implications for health, more work is needed to understand barriers and identify methods to address them to improve awareness among racial and ethnic minority communities.

In addition to identifying racial, ethnic, and income disparities, this analysis identified differences in awareness of genetic testing by cancer history. Notably, respondents with a family history of cancer were more likely to be aware of cancer genetic testing than those without a family history of cancer, and respondents with a personal history of cancer were more likely to have cancer genetic testing than those without a personal history of cancer. This is consistent with the findings from previous HINTS iterations^1,10^ and previous work using HINTS 5 cycle 4, which found that those with a history of breast, ovarian or colorectal cancer were more likely to have cancer genetic testing.^5^ In this analysis, results may have been driven by breast cancer cases among those with a personal history of cancer. Although these results are encouraging that there is an increased awareness of genetic testing for those with a family history or personal history of cancer, we were unable to confirm that those reporting of having a genetic test did so in accordance with current clinical care guidelines.

Although a strength of the study is the nationally representative nature of the survey, the response rate was low (37%). However, of note, weighted responses were similar to unweighted values. Second, because HINTS is a cross-sectional study, we are unable to comment on the temporal relationship between awareness and receipt of genetic testing in our study population. Another limitation is that some demographic groups were not well represented, including those with lower education and without health insurance. This limited our ability to examine association by these characteristics and limited generalizability. In addition, we were unable to examine other types of clinical genetic testing besides cancer testing (eg, paternity testing, prenatal screening). The absence of detailed information on personal and family history of cancer also limited the ability to evaluate in accordance with clinical guidelines. Because there were small number of individuals who had a personal history of cancer, we were unable to look at the differences across different cancer types. In addition, there were a small number of individuals with a personal history of cancer and who had cancer genetic testing, limiting our ability to evaluate the relationship between these variables. Future work is needed to examine uptake of genetic testing in those populations in which genetic testing is advised on the basis of clinical guidelines. Finally, in this analysis, we analyzed awareness of genetic testing, but we did not assess understanding of genetic testing among respondents.

The results of this analysis suggest that most Americans are aware of genetic testing, but differences by race, ethnicity, and income persist for both awareness and use of genetic testing overall and by the different test types. Because of the increasing importance of genetic testing in the health care setting, it is critical to assess and address barriers to both awareness and access to genetic testing.

## Supplementary Material

1

## Figures and Tables

**Table 1:** Demographic characteristics for HINTS 5 cycle 4 respondents (*N* = 3767)

Characteristic	*n*	Wgt %^a^

Sex		
Male	1561	49
Female	2206	51
Age, y		
18–34	500	26
35–49	722	26
50–64	1152	28
65–74	862	12
≥75	531	8
Race/ethnicity		
Non-Hispanic White	2268	60
Non-Hispanic Black	569	15
Non-Hispanic Asian	174	5
Non-Hispanic American Indian, Alaska Native, Pacific Islander	29	1
Non-Hispanic multiracial	108	3
Hispanic	619	16
Level of education		
High school or less	973	30
Some college	2794	70
Employment status^b^		
Employed	1852	59
Retired	1165	19
Disabled	201	5
Other	298	12
Not employed	130	4
Health insurance status		
Insured	3568	91
Not insured	199	9
Income^c^		
Less than $20,000	609	15
$20,000 to $49,999	890	24
$50,000 to $99,999	982	31
$100,000 or higher	909	30
Marital status		
Married	2010	55
Other^d^	1104	14
Never married	653	31
Personal history of cancer		
Yes	464	7
No	3303	93
Type of cancer		
Breast cancer	105	19
Prostate cancer	84	9
Other^e^	275	72
Family history of cancer^c^		
Yes	2630	70
No	720	22
Not Sure	308	8

*HINTS*, Health Information National Trends Survey; *Wgt*, weighted.

a These were calculated using jackknife replication.

b Participants who reported of being employed full or part time were categorized as “employed.” Participants who were retired, disabled, students, homemakers, or other were categorized as “not employed.” *n* = 121 participants were missing responses for employment.

c *n* = 377 participants were missing responses for income, and 109 were missing responses for family history of cancer.

d The “other” category included participants who were widowed, divorced, or separated.

e Participants belonging to the “other” cancer type category included those with bladder cancer, bone cancer, cervical cancer, colon cancer, endometrial cancer, head/neck cancer, Hodgkin’s lymphoma, renal cancer, leukemia, liver cancer, lung cancer, non-Hodgkin’s, oral cancer, ovarian cancer, pancreatic cancer, pharyngeal cancer, rectal cancer, melanoma, or another type of cancer.

**Table 2:** Demographic and cancer history for awareness of and receipt of genetic testing overall

	Awareness of Genetic Testing (*N* = 3767)			Receipt of Genetic Testing (*N* = 3648)		
Characteristic	Yes *n* (Wgt %^a^)	No *n* (Wgt %^a^)	Adjusted Odds Ratio^b^ (95% CI)	Yes *n* (Wgt %^a^)	No *n* (Wgt %^a^)	Adjusted Odds Ratio^b^ (95% CI)

Sex	1166 (76)	395 (24)	Ref	258 (13)	1251 (87)	Ref
Male						
Female	1665 (77)	541 (23)	1.1 (0.7–1.6)	463 (22)	1676 (78)	2.0 (1.4–2.7)
Age, y						
18–34	415 (80)	85 (20)	Ref	96 (14)	400 (86)	Ref
35–49	574 (78)	148 (22)	0.8 (0.4–1.6)	139 (17)	567 (83)	1.1 (0.7–1.8)
50–64	878 (78)	274 (22)	0.8 (0.4–1.4)	223 (21)	904 (79)	1.2 (0.8–2.0)
65–74	635 (73)	227 (27)	0.6 (0.3–1.1)	169 (19)	655 (81)	1.2 (0.7–2.1)
≥75	329 (60)	202 (40)	0.4 (0.2–0.8)	94 (16)	401 (84)	1.1 (0.6–2.0)
Race/ethnicity						
Non-Hispanic White	1882 (83)	386 (17)	Ref	51,569 (23)	1682 (77)	Ref
Non-Hispanic Black	374 (66)	195 (34)	0.6 (0.4–1.0)	76 (14)	471 (86)	0.5 (0.3–0.9)
Non-Hispanic Asian	102 (59)	72 (41)	0.2 (0.1–0.5)	22 (13)	151 (87)	0.7 (0.3–1.4)
Non-Hispanic other^c^	113 (82)	24 (18)	1.3 (0.7–2.6)	37 (27)	99 (73)	1.7 (0.8–3.7)
Hispanic	360 (58)	259 (42)	0.4 (0.3–0.7)	71 (12)	524 (88)	0.4 (0.2–0.6)
Level of education						
High school or less	530 (61)	443 (39)	Ref	112 (12)	816 (88)	Ref
Some college	2301 (83)	493 (17)	2.3 (1.5–3.4)	609 (20)	2111 (80)	1.4 (0.9–2.1)
Employment status^d^						
Employed	1497 (81)	355 (19)	Ref	369 (19)	1450 (81)	Ref
Not Employed	1265 (71)	529 (29)	1.0 (0.7–1.5)	335 (16)	1380 (84)	0.9 (0.6–1.2)
Income^e^						
Less than $20,000	348 (61)	261 (39)	Ref	63 (10)	520 (90)	Ref
$20,000-$49,999	621 (67)	269 (33)	1.2 (0.8–1.9)	139 (15)	714 (85)	1.5 (0.8–2.6)
$50,000-$99,999	818 (82)	164 (18)	2.2 (1.3–3.8)	220 (21)	739 (79)	2.0 (1.2–3.4)
$100,000 or higher	803 (88)	106 (12)	2.9 (1.6–5.2)	244 (22)	653 (78)	1.9 (1.2–3.3)
Marital status						
Married	1585 (79)	425 (21)	Ref	427 (21)	1526 (79)	Ref
Other^f^	757 (69)	347 (31)	0.9 (0.6–1.4)	190 (17)	868 (83)	0.9 (0.6–1.3)
Never married	489 (76)	164 (24)	1.0 (0.7–1.6)	104 (12)	533 (88)	0.8 (0.5–1.2)
Personal history of cancer						
No	2506 (77)	797 (23)	Ref	610 (17)	2590 (83)	Ref
Yes	325 (75)	139 (25)	0.9 (0.6–1.4)	111 (24)	337 (76)	1.6 (1.0–2.7)
Family history of cancer^e^						
No	475 (68)	245 (32)	Ref	102 (12)	602 (88)	Ref
Yes	2110 (81)	520 (19)	1.6 (1.0–2.7)	561 (20)	1988 (80)	1.4 (0.9–2.0)
Not sure	177 (62)	131 (38)	1.0 (0.6–2.0)	43 (12)	253 (88)	1.2 (0.6–2.4)

*Ref*, reference group; *Wgt*, weighted.

a These were calculated using jackknife replication.

b Odds ratios were adjusted for the other characteristics shown in this table.

c Those identifying as Alaska Native/American Indian, Pacific Islander, or of multiple races were combined into an “other” race category owing to low cell counts.

d Participants who reported of being employed full or part time were categorized as “employed”. Participants who were retired, disabled, students, homemakers, or other were categorized as “not employed.” *n* = 121 participants were missing responses for employment.

e *n* =377 participants were missing responses for income, and 109 were missing responses for family history of cancer.

f The “other” category included participants who were widowed, divorced, or separated.

**Table 3:** Adjusted analysis for awareness of genetic testing by different test types

	Ancestry (*N* = 3767)			Health (*N* = 3767)			Cancer (*N* = 3767)		
Characteristic	Yes *n* (Wgt %^a^)	No *n* (Wgt %^a^)	Adjusted Odds Ratio^b^ (95% CI)	Yes *n* (Wgt %^a^)	No *n* (Wgt %^a^)	Adjusted Odds Ratio^b^ (95% CI)	Yes *n* (Wgt %^a^)	No *n* (Wgt %^a^)	Adjusted Odds Ratio^b^ (95% CI)

Sex									
Male	1118 (72)	443 (28)	Ref	786 (52)	775 (48)	Ref	439 (30)	1122 (70)	Ref
Female	1574 (73)	632 (27)	1.1 (0.8–1.6)	1215 (57)	991 (43)	1.4 (1.0–1.9)	917 (42)	1289 (58)	2.0 (1.4–2.7)
Age, y									
18–34	403 (76)	97 (24)	Ref	338 (61)	162 (39)	Ref	195 (34)	305 (66)	Ref
35–49	535 (72)	187 (28)	0.7 (0.4–1.4)	452 (60)	270 (40)	0.8 (0.4–1.4)	332 (43)	390 (57)	1.4 (0.9–2.3)
50–64	842 (75)	310 (25)	0.9 (0.5–1.5)	597 (50)	555 (50)	0.5 (0.3–0.8)	431 (37)	721 (63)	1.0 (0.7–1.6)
65–74	602 (71)	260 (29)	0.7 (0.3–1.5)	442 (52)	420 (48)	0.5 (0.3–0.8)	283 (31)	579 (69)	0.8 (0.4–1.3)
>75	310 (57)	221 (43)	0.6 (0.3–1.1)	172 (34)	359 (66)	0.3 (0.2–0.5)	115 (22)	416 (78)	0.6 (0.3–1.1)
Race/ethnicity									
Non-Hispanic White	1813 (80)	455 (20)	Ref	1416 (62)	852 (38)	Ref	953 (42)	1313 (58)	Ref
Non-Hispanic Black	347 (61)	222 (39)	0.6 (0.4–0.9)	219 (38)	350 (62)	0.6 (0.4–0.9)	150 (26)	419 (74)	0.5 (0.3–0.8)
Non-Hispanic Asian	89 (51)	85 (49)	0.2 (0.09–0.4)	77 (44)	97 (56)	0.4 (0.3–0.8)	48 (28)	126 (72)	0.7 (0.3–1.3)
Non-Hispanic other^c^	108 (79)	29 (21)	0.9 (0.3–2.4)	79 (58)	58 (42)	0.8 (0.4–1.8)	61 (44)	76 (55)	1.0 (0.4–2.9)
Hispanic	335 (54)	284 (46)	0.4 (0.3–0.7)	210 (34)	409 (66)	0.4 (0.2–0.7)	144 (23)	475 (77)	0.5 (0.3–0.8)
Level of education									
High school or less	485 (56)	488 (44)	Ref	277 (35)	696 (65)	Ref	185 (22)	788 (78)	Ref
Some college	2207 (79)	587 (21)	2.3 (1.5–3.3)	1724 (63)	1070 (37)	2.3 (1.6–3.3)	1171 (42)	1623 (58)	1.9 (1.3–2.8)
Employment status^d^									
Employed	1441 (77)	411 (23)	Ref	1144 (60)	708 (40)	Ref	790 (39)	1062 (61)	Ref
Not employed	1188 (66)	606 (34)	0.8 (0.6–1.2)	814 (48)	980 (52)	1.1 (0.7–1.5)	540 (31)	1254 (69)	1.0 (0.7–1.6)
Income^e^									
Less than $20,000	314 (54)	295 (46)	Ref	204 (36)	405 (64)	Ref	133 (22)	476 (78)	Ref
$20,000-$49,999	584 (64)	306 (36)	1.4 (0.9–2.1)	389 (42)	501 (58)	1.2 (0.8–1.8)	260 (28)	630 (72)	1.2 (0.8–1.9)
$50,000-$99,999	791 (79)	191 (21)	2.3 (1.2–4.2)	594 (62)	388 (38)	2.1 (1.4–3.1)	398 (40)	584 (60)	1.7 (1.2–2.5)
$100,000 or higher	777 (84)	132 (16)	2.6 (1.5–4.6)	659 (67)	250 (33)	2.1 (1.2–3.6)	463 (47)	446 (53)	2.0 (1.2–3.4)
Marital status									
Married	1520 (75)	490 (25)	Ref	1176 (59)	834 (41)	Ref	796 (40)	1214 (60)	Ref
Other^f^	710 (65)	394 (35)	0.9 (0.6–1.3)	473 (41)	631 (59)	0.8 (0.5–1.1)	336 (30)	768 (70)	0.9 (0.6–1.2)
Never married	462 (71)	191 (29)	1.0 (0.7–1.6)	352 (53)	301 (47)	0.8 (0.5–1.2)	224 (32)	429 (68)	1.0 (0.7–1.5)
Personal history of cancer									
No	2387 (73)	916 (27)	Ref	1791 (54)	1512 (46)	Ref	1199 (36)	2104 (64)	Ref
Yes	305 (68)	159 (32)	0.9 (0.6–1.4)	210 (54)	254 (46)	1.1 (0.8–1.6)	157 (37)	307 (63)	1.2 (0.8–1.7)
Family history of cancer^e^									
No	448 (64)	272 (36)	Ref	310 (45)	410 (55)	Ref	196 (28)	524 (72)	Ref
Yes	2011 (76)	619 (24)	1.5 (0.9–2.3)	1535 (58)	1095 (42)	1.6 (1.1–2.4)	1079 (41)	1551 (59)	1.8 (1.3–2.5)
Not sure	169 (59)	139 (41)	1.2 (0.6–2.1)	102 (44)	206 (56)	1.5 (0.8–3.0)	49 (16)	259 (84)	0.7 (0.4–1.5)

*Ref*, reference group; *Wgt*, weighted.

a These were calculated using jackknife replication.

b Odds ratios were adjusted for the other characteristics shown in this table.

c Those identifying as Alaska Native/American Indian, Pacific Islander, or of multiple races were combined into an “other” race category owing to low cell counts.

d Participants who reported of being employed full or part time were categorized as “employed.” Participants who were retired, disabled, students, homemakers, or other were categorized as “not employed.” *n* = 121 participants were missing responses for employment.

e *n* =377 participants were missing responses for income, and 109 were missing responses for family history of cancer._”_

f The “other” category included participants who were widowed, divorced, or separated.

**Table 4:** Adjusted analysis for receiving genetic testing by different test types

	Ancestry (*N* = 3648)			Health (*N* = 3648)			Cancer (*N* = 3648)		
Characteristic	Yes *n* (Wgt %^a^)	No *n* (Wgt %^a^)	Adjusted Odds Ratio^b^ (95% CI)	Yes *n* (Wgt %^a^)	No *n* (Wgt %^a^)	Adjusted Odds Ratio^b^ (95% CI)	Yes *n* (Wgt %^a^)	No *n* (Wgt %^a^)	Adjusted Odds Ratio^b^ (95% CI)

Sex									
Male	221 (11)	1288 (89)	Ref	76 (4)	1433 (96)	Ref	32 (2)	1477 (98)	Ref
Female	317 (15)	1822 (85)	1.4 (1.0–2.1)	168 (7)	1971 (93)	2.1 (1.4–3.2)	113 (5)	2026 (95)	3.5 (1.9–6.2)
Age, y									
18–34	75 (12)	421 (88)	Ref	50 (6)	446 (94)	Ref	10 (1)	486 (99)	Ref
35–49	87 (11)	619 (89)	0.8 (0.5–1.4)	58 (6)	648 (94)	0.7 (0.4–1.5)	33 (4)	673 (96)	3.0 (1.0–9.4)
50–64	162 (15)	965 (85)	1.1 (0.7–1.9)	70 (6)	1057 (94)	0.8 (0.4–1.4)	55 (6)	1072 (94)	3.2 (0.9–10.9)
65–74	135 (15)	689 (85)	1.3 (0.7–2.3)	43 (5)	781 (95)	0.8 (0.3–2.0)	31 (3)	793 (97)	1.4 (0.3–6.6)
>75	79 (14)	416 (87)	1.5 (0.8–3.0)	23 (4)	472 (96)	0.7 (0.2–2.5)	16 (3)	479 (97)	1.4 (0.2–8.7)
Race/ethnicity									
Non-Hispanic White	387 (18)	1810 (82)	Ref	171 (8)	2026 (92)	Ref	95 (4)	2102 (96)	Ref
Non-Hispanic Black	52 (10)	495 (90)	0.5 (0.3–0.9)	24 (4)	523 (96)	0.9 (0.4–2.0)	21 (4)	526 (96)	1.2 (0.6–2.6)
Non-Hispanic Asian	17 (10)	156 (90)	0.7 (0.3–1.8)	12 (7)	161 (93)	1.5 (0.5–4.6)	2 (1)	171 (99)	0.2 (0.01–3.1)
Non-Hispanic Other^c^	26 (19)	110 (81)	1.5 (0.6–3.5)	16 (12)	120 (88)	1.6 (0.6–3.9)	9 (7)	127 (93)	3.2 (0.4–23.6)
Hispanic	56 (9)	539 (91)	0.4 (0.3–0.7)	21 (5)	574 (96)	0.3 (0.1–0.9)	18 (3)	577 (97)	0.5 (0.2–1.0)
Level of education									
High school or less	73 (7)	855 (93)	Ref	40 (4)	888 (96)	Ref	35 (4)	893 (96)	Ref
Some college	465 (15)	2255 (85)	1.9 (1.2–2.9)	204 (7)	2516 (93)	1.3 (0.6–2.9)	110 (4)	2610 (96)	0.7 (0.5–1.7)
Employment status^d^									
Employed	267 (14)	1552 (86)	Ref	142 (7)	1677 (93)	Ref	77 (4)	1742 (96)	Ref
Not employed	261 (12)	1454 (88)	0.9 (0.6–1.3)	95 (4)	1620 (96)	0.6 (0.4–1.0)	66 (4)	1649 (96)	1.1 (0.5–2.2)
Income^e^									
Less than $20,000	37 (6)	546 (94)	Ref	30 (3)	553 (97)	Ref	20 (3)	563 (97)	Ref
$20,000-$49,999	106 (11)	747 (89)	1.7 (0.8–3.5)	47 (5)	806 (95)	1.6 (0.6–4.2)	28 (2)	825 (98)	0.7 (0.3–2.0)
$50,000-$99,999	175 (16)	784 (84)	2.4 (1.2–5.0)	59 (5)	900 (95)	1.4 (0.6–3.1)	39 (4)	920 (96)	2.0 (0.8–5.0)
$100,000 or higher	178 (15)	719 (85)	2.0 (0.9–4.3)	96 (8)	801 (92)	2.0 (1.0–4.1)	47 (5)	850 (95)	2.2 (1.0–4.9)
Marital status									
Married	319 (15)	1634 (85)	Ref	143 (7)	1810 (93)	Ref	75 (4)	1878 (96)	Ref
Other^f^	138 (13)	920 (87)	0.9 (0.6–1.4)	69 (6)	989 (94)	1.1 (0.7–1.9)	52 (5)	1006 (95)	1.3 (0.6–2.7)
Never married	81 (10)	556 (90)	0.8 (0.5–1.3)	32 (4)	605 (96)	0.6 (0.3–1.3)	18 (2)	619 (98)	0.8 (0.3–2.5)
Personal history of cancer									
No	476 (13)	2724 (87)	Ref	217 (6)	2983 (94)	Ref	98 (3)	3102 (97)	Ref
Yes	62 (11)	386 (89)	0.8 (0.4–1.4)	27 (6)	421 (94)	1.2 (0.6–2.1)	47 (14)	401 (86)	5.3 (2.7–10.3)
Family history of cancer^e^									
No	83 (9)	621 (91)	Ref	36 (6)	668 (94)	Ref	9 (1)	695 (99)	Ref
Yes	407 (15)	2142 (85)	1.5 (1.0–2.2)	186 (6)	2363 (94)	0.9 (0.4–1.8)	125 (5)	2424 (95)	2.5 (0.8–8.3)
Not Sure	38 (11)	258 (89)	1.8 (0.8–3.9)	16 (5)	280 (95)	1.2 (0.3–4.5)	7 (1)	289 (99)	0.7 (0.1–4.1)

*Ref*, reference group; *Wgt*, weighted.

a These were calculated using jackknife replication.

b Odds ratios were adjusted for the other characteristics shown in this table.

c Those identifying as Alaska Native/American Indian, Pacific Islander, or of multiple races were combined into an “other” race category owing to low cell counts.

d Participants who reported of being employed full or part time were categorized as “employed.” Participants who were retired, disabled, students, homemakers, or other were categorized as “not employed.” *n* = 121 participants were missing responses for employment.

e *n* =377 participants were missing for income, and 109 were missing responses for family history of cancer.

f The “other” category included participants who were widowed, divorced, or separated.

## Data Availability

The data used in this analysis is available to the public at https://hints.cancer.gov/data/default.aspx
