# Supplementary material for: Awareness and use of genetic testing: An analysis of the Health Information National Trends Survey 2020
Source: Genet Med. Author manuscript; Available in PMC 2022 Dec 13. (PMC9746668; doi:10.1016/j.gim.2022.08.023)
Supplement: 1 [file NIHMS1841636-supplement-1.pdf]

Supplementary Table I. Demographic characteristics for HINTS 5 Cycle 4 Respondents with weighted and unweighted percentages (*N* = 3,767).

Characteristic	*n*	Weighted[a] %	Unweighted %
**Sex**			
Male	1,561	49	41
Female	2,206	51	59
**Age**			
18-34	500	26	13
35-49	722	26	19
50-64	1,152	28	31
65-74	862	12	23
75 and above	531	8	14
**Race**			
Non-Hispanic White	2,268	63	60
Non-Hispanic Black	569	12	15
Non-Hispanic Asian	174	5	5
Non-Hispanic American Indian, Alaska Native, Pacific Islander	29	1	1
Non-Hispanic Multiracial	108	2	3
Hispanic	619	16	16
**Level of Education**			
High School or less	1,014	30	26
Some College	2,851	70	74
**Employment Status[b]**			
Employed	1,852	59	51
Retired	1,165	19	32
Disabled	201	5	6
Other	298	12	8
Not Employed	130	4	4
**Health Insurance Status**			
Insured	3,568	91	95
Not Insured	199	9	5
**Income[c]**			
Less than $20,000	609	15	18
$20,000 to $49,999	890	24	26
$50,000 to $99,999	982	31	29
$100,000 or more	909	30	27
**Marital Status**			
Married	2,010	55	53
Other[d]	1,104	14	29
Never Married	653	31	17
**Personal History of Cancer**			
Yes	462	7	12
Breast Cancer	105	19	23
Prostate Cancer	84	9	18
Other[e]	275	72	59
No	3,305	93	88
**Family History of Cancer[c]**			
Yes	2,630	70	72

No	720	22	20
Not Sure	308	8	8

[a] Weighted percentages. These were calculated using jackknife replication.
[b] Participants who reported being employed full or part time were categorized as “employed”. Participants who were retired, disabled, students, homemakers or other were categorized as “not employed.” *n* = 121 participants were missing responses for employment.
[c] *n* = 377 participants were missing for income and 109 were missing responses for family history of cancer.
[d] The “other” category included participants who were widowed, divorced, or separated.
[e] Participants belonging to the “other” cancer type category included those with bladder cancer, bone cancer, cervical cancer, colon cancer, endometrial cancer, head/neck cancer, Hodgkin’s Lymphoma, renal cancer, leukemia, liver cancer, lung cancer, non-Hodgkin’s, oral cancer, ovarian cancer, pancreatic cancer, pharyngeal cancer, rectal cancer, melanoma, or another type of cancer.
